# PPAR**β/δ** a potential target in pulmonary hypertension blighted by cancer risk

**DOI:** 10.1177/2045894018812053

**Published:** 2018-10-23

**Authors:** Jane A. Mitchell, David Bishop-Bailey

**Affiliations:** 1 Cardiothoracic Pharmacology, National Heart and Lung Institute, Imperial College, London, UK; 2 Comparative Biomedical Sciences, Royal Veterinary College, London, UK

Dear Editor,

Our group and others have used preclinical in vitro and in vivo models that highlight the potential therapeutic benefit of PPARβ/δ as a target in the treatment of pulmonary arterial hypertension (PAH). Selective agonists of PPARβ/δ inhibit fibroblast and pulmonary arterial vascular smooth muscle cell growth and prevent right heart hypertrophy in rat models of PAH. Further work published in *Pulmonary Circulation* established the transcriptomic profile and pathways associated with activating PPARβ/δ in a model of pulmonary artery banding and right heart hypertrophy.^1^ These results and the fact that enhancing PPARβ/δ is linked to increased endurance exercise performance^2^ supports the idea that drugs working on this pathway could be beneficial in PAH. However, there is cause for concern regarding at least one drug that activates PPARβ/δ, GW501516, developed by GlaxoSmithKline plc (GSK) in the early 2000s. Despite these concerns and although not confirmed in humans, following the publication of endurance exercise studies in rodents, a significant underground market has developed for unlicensed GW501516 (also referred to as Endurobol or Cardarine) in a bid to enhance human athletic performance.

PPARβ/δ agonists, including GW501516, were developed for the treatment of hyperlipidemia and other cardiovascular diseases; a number of clinical trials have been registered on clinicaltrials.gov (NCT00388180, NCT00318617, NCT00158899, NCT00841217). While long-term clinical data are not available, GW501516 improved lipid profiles in short-term studies in man.^3–5^ However safety concerns over GW501516 and potentially other drugs in the class have emerged. Of particular relevance are two abstracts from GSK showing that GW501516 causes cancer in rats^6^ and mice^7^ after 104 weeks of dosing. Although neither of these studies has been published as full peer-reviewed papers, these abstracts have been very influential. The phase 4 trial (NCT00841217) was stopped and warnings issued by the World Anti-Doping Agency,^8^ Health Canada,^9^ and, most recently in April 2018, GW501516 was classified as a poisonous substance in Australia.^10^ The precise role of PPARβ/δ in cancer, particularly in humans, however, remains unclear as reports continue to emerge showing that agonists may either increase or protect against different cancers.^11^

Despite these controversies, PPARβ/δ remains a potentially important therapeutic target for the future treatment of PAH. Now more research needs to be conducted to fully understand the carcinogenic (and other) side effects of drugs that activate PPARβ/δ before they can be translated into therapies to treat long-term chronic diseases such as PAH. In particular, once detrimental pathways can be distinguished from protective pathways, it would be of great interest to investigate whether selective modulators exist or could be developed that specifically target PAH while sparing any pro-carcinogenic activity.
